# Wild Heterotrophic Nitrifying Strain *Pseudomonas* BT1 Isolated from Kitchen Waste Sludge Restores Ammonia Nitrogen Removal in a Sewage Treatment Plant Shocked by Thiourea

**DOI:** 10.1007/s12010-022-03850-7

**Published:** 2022-03-16

**Authors:** Jingxuan Deng, Zhenxing Huang, Jinbo Wang, Xiaohong Shan, Wansheng Shi, Wenquan Ruan

**Affiliations:** 1 School of Environment and Civil Engineering, Jiangnan University, Wuxi, 214122 China; 2 Jiangsu Key Laboratory of Anaerobic Biotechnology, Jiangnan University, Wuxi, 214122 China; 3 JiangNing Water Group Co., Ltd, Nanjing, 211100 China; 4 Wuxi MaSun Environmental Energy Technology Co., Ltd, Wuxi, 214122 China

**Keywords:** Heterotrophic nitrification, Thiourea, BT1

## Abstract

Thiourea is used in agriculture and industry as a metal scavenger, synthetic intermediate, and nitrification inhibitor. However, in wastewater, it can inhibit the nitrification process and induce the collapse of the nitrification system. In such a case, ammonia-oxidizing bacteria (AOB) lose their ability to remove ammonia. We investigated the nitrification system of a 60,000-t/d municipal sewage treatment plant in Nanjing, which collapsed after receiving 5–15 ppm (5–15 mg/L) thiourea. Ammonia nitrogen removal quickly recovered to more than 95% after inoculation with 10 t high-efficiency nitrification sludge, which was collected from a kitchen waste treatment plant. A heterotrophic nitrification strain was isolated from the inoculated sludge and identified as wild *Pseudomonas* by 16S rDNA sequencing and named “BT1.” Based on thiourea tolerance tests, BT1 can tolerate a thiourea content of more than 500 ppm. For comparison, the in situ process was imitated by the simulation system, and the wastewater shocked by 10 ppm thiourea could still meet the emission standard after adding 1% (V/V) BT1. High-throughput sequencing analysis was applied to study microbial succession during thiourea shock loading. The results showed that *Hydrogenophaga* and *Thiobacillus* grew with the growth of BT1. *Pseudomonas* BT1 was used for a 6,000-t/d printed circuit board (PCB) wastewater treatment system, the nitrification system returned to normal in 15 days, and the degradation rate stabilized at more than 95%.

**Supplementary Information:**

The online version contains supplementary material available at 10.1007/s12010-022-03850-7.

## Introduction

Thiourea is widely used in various industries [1, 19, 22], for example, as a metal scavenger in the metallurgical industry and in printed metal board manufacturing [17, 23]) and as a synthetic intermediate in the pharmaceutical and resin industries [10, 16]. These industries often generate large amounts of thiourea-containing wastewater [11]. In sewage treatment plants, thiourea inhibits nitrification,it is a specific nitrification inhibitor because its C=S structure can covalently bond with copper in the active center of ammonia-oxidizing bacteria (AOB), causing them to lose their ammonia oxidation ability [30, 31]. In agriculture, thiourea is frequently used as a nitrification inhibitor to inactivate ammonia-oxidizing bacteria and delay urea hydrolysis [12, 36]. The toxicity of thiourea to the nitrification system has been reported for activated sludge, and even at low concentrations, thiourea can have significant impacts on sewage treatment systems [20].

If the nitrification system of a sewage treatment plant is severely impacted, the addition of AOB, including autotrophic nitrifying bacteria (ANB) and heterotrophic nitrifying bacteria (HNB), can facilitate the restoration of nitrogen removal, helping the system recover rapidly [14, 38, 39], and this approach has been widely accepted [33, 35]. Boon et al. [3] reported that the inhibitory effects of 3-chloroaniline on nitrification were successfully eliminated by inoculating 3-chloroaniline-degrading bacteria, which also recovered ammonium-nitrogen removal [3]. ANB usually grow slowly, contain fewer species, are difficult to cultivate, and are sensitive to toxicity, whereas HNB grow faster and have more varieties. In addition, the costs of ANB are high, mainly because of the complex separation and purification processes. Most studies on HNB have focused on reducing the C/N ratio, denitrification, and low-temperature ammonia oxidation [13], [21], [32],however, the specificity of HNB in engineering applications is low. In this sense, HNB can also be used to protect specific ANB from toxicity, which is important to restore the nitrification efficiency of sewage treatment plants. Due to the limitations of the AOB that have been discovered, a strain of bacteria with low producing cost and high specificity is urgent and important for sewage treatment systems to restore ammonia nitrogen removal after the collapse.

In a full-scale wastewater treatment application, we used the bioaugmentation method to inoculate high-efficiency nitrification sludge obtained from a high-efficiency kitchen waste treatment plant and introduced into a 60,000 t/d sewage treatment plant in Nanjing that had collapsed under 5–15 ppm thiourea; the sludge adding ratio was more than 0.15‰, and the nitrification system could be successfully restored. To study the strains involved, we isolated and purified HNB from the inoculated sludge and designed a continuous simulator to recreate thiourea shock, nitrification system collapse, HNB enhancement, and system recovery. We performed high-throughput sequencing analysis at each stage to further investigate the mechanisms underlying HNB bioaugmentation.

## Materials and Methods

### Restoration of Nitrification Ability of a 60,000 T/d Plant by Kitchen Sludge

The influent investigated here was composed of treated industrial sewage (approximately 30%) from more than 400 chemical factories and domestic sewage (approximately 70%). The system was shocked in mid-February 2018, which resulted in a significantly reduced nitrification removal rate (to approximately 50%). Sludge samples from the sewage were collected for microscopic examination. The thiourea content in the influent and effluent water was also measured.

The investigated kitchen sewage treatment system is a high-efficiency nitrification and denitrification system. The parameters of the high-efficiency DN (denitrification and nitrification) reactor from the kitchen wastewater process are shown in Table 1. Sludge from the kitchen sewage treatment system was collected and inoculated into the shocked treatment system at a ratio of 10:60,000 t (more than 0.15‰). The thiourea content in the influent and effluent water and NH4+-N level were monitored according to the national standard method [2].

**Table 1:** Parameters of the high-efficiency DN reactor from kitchen wastewater process

NH_4_-N INF (ppm)	NH_4_-N EFF (ppm)	TN-INF (ppm)	TN-EFF (ppm)	COD-INF (ppm)	COD-EFF (ppm)	MLSS (ppm)	DO (ppm)	RAS	SV30	HRT (h)
2000 ± 200	20–35	2,500 ± 500	200–300	8,000 ± 2,000	700–1,000	8,000–10,000	<1	1000%	100%	36–48

### Strain isolation, Purification, and Identification

Thiourea-resistant bacteria and HNB were isolated and purified using the traditional culture medium separation method. A total of 100 μL sludge was collected from the high-efficiency nitrification reactor of a kitchen waste treatment system and diluted to 10^−5^ and 10^−6^ ppm by adding 10 ppm thiourea solution. Subsequently, we added Gris color reagent to perform flat coating, and the bacteria were cultured at 30 °C in a biological incubator for 24–48 h. Then, a single plaque in the pink area was selected using the sterilized loop, and purified by lining separation repeatedly. Pick an isolated colony and spread it over the first quadrant (approximately 1/4 of the plate) using close parallel streaks, and then immediately streak the inoculating loop very gently over a quarter of the plate using a back-and-forth motion. Sterilize the loop and allow it to cool. Repeat the pervious step for third times to extend the streaks to the whole plate. The isolated strains continued to expand cultivation for preparation. The isolation medium was ammonium organic solid medium (NH_4_Cl 0.47 g L^−1^, sodium succinate 5.62 g L^−1^, trace element solution 50 mL L^−1^, and agar 15 g L^−1^) [9]). The trace element solution contained K_2_HPO_4_ (5,000 mg L^−1^), MgSO_4_·7H_2_O (2,500 mg L^−1^), NaCl (2,500 mg L^−1^), FeSO_4_·7H_2_O (50 mg L^−1^), and MnSO_4_·4H_2_O (50 mg L^−1^). The expanded cultivation medium was ammonium organic liquid medium, which was the same as the isolation medium except without agar. Expanded cultivation was performed at 30 °C in 250 mL flasks, which were shaken at 160 rpm until an optical density at 600 nm (OD600) of 0.8–1.0 was reached, followed by centrifugation (4,000×*g*, 10 min). The obtained sediment was used for species identification by 16S rDNA sequencing. Physiological and biochemical analyses of the culture solution were performed to determine the nitrification ability and the growth rate of bacteria,the obtained strain was stored with 20% glycerol at −80 °C for further analysis.

### Strain Thiourea Tolerance Test

The purified strain grew under aerobic conditions in 100 ml ammonium organic liquid medium at 30 °C in 250 ml flasks shaken at 160 rpm until an optical density at 600 nm (OD600) was reached 0.8–1.0. The cells were then centrifuged (4000×*g*, 10 min) and resuspended in the same volume of ammonium organic medium containing 0, 10, 100, 250, 350, and 500 mg/L thiourea. Then, the cells were grown in triplicate and shaken under the same conditions for 48 h. NH_4_^+^-N and cell concentration (OD600) were measured every 12 h during the cultivation cycles.

### Continuous Reaction Simulator

Two continuous reactors (reactor A and reactor B), each with a working volume of 5.0 L, were designed (29 cm internal diameter × 65 cm height) (Supplementary Fig. 1). The reactors were made of polymethyl methacrylate (PMMA) and were equipped with a control station (BioFlo 320, Eppendorf, Germany), which was connected with a pH and DO meter (PO720, Mettler Toledo, Switzerland), a mechanical stirrer (PO480, Germany), an aeration sparger, and an air pump (GA61, Greeloy, China). The operational temperature was maintained at 24 ± 1 °C via a heated water bath using a temperature controller connected to the control station.

Seeding sludge was collected from an A/O biochemical sedimentation tank of a 60,000-t/d sewage treatment plant in Nanjing, China. The seeding sludge was equally injected into the reactors, followed by mixing with wastewater. The concentration of mixed liquid suspended solids (MLSS) was 4,000 ± 100 mg/L in both reactors. The wastewater used in this study was synthetic and was composed of NH_4_Cl and sodium acetate as the main sources of ammonium and organic carbon, respectively. The concentrations of ammonium and organic carbon were 96 mg L^−1^ and 512.5 mg L^−1^, respectively. The other components included NaHCO_3_ (287 ppm), MgSO_4_·7H_2_O (50 ppm), CaCl_2_·2H_2_O (28 ppm), KH_2_PO_4_ (6.6 ppm), K_2_HPO_4_ (8.4 ppm), and EDTA (10 ppm). The pH was adjusted to 7.5–8.0. The sludge of municipal sewage is generally used for about 12–15 day, so the seeding sludge was pretreated with synthetic wastewater for 1 week to keep the activity and diversity of the sludge to adapt the simulation system. Then, the sludge after pretreatment provided a favorable environment prior to the thiourea shock experiment. Two reactors were operated with continuous feeding, aerating, stirring, and decanting. During the decanting phase, 4 L of supernatant was discharged from the reactor, and activated sludge precipitation refluxed to the reactor to maintain the MLSS concentration at 3500–4000 mg VSS L^−1^ during the entire operational period, which was 21 days. The hydraulic retention time (HRT) of the influent was 12 h, and during the 21 days, there was no sludge discharge. Chemical oxygen demand (COD), NH4+-N, and MLSS levels were measured according to standard methods [2]. The ammonia oxide rate (AOR) of the reactor was calculated by Equation (1) [2]. At an ammonia removal rate below 80%, the ammonia in the effluent exceeds the standard of the actual sewage treatment system.

1 $$\mathrm{Ammonia oxide rate}=\frac{(\mathrm{Inf}.{{\mathrm{NH}}_{4}}^{+}-\mathrm{N})-(\mathrm{Eff}.{{\mathrm{NH}}_{4}}^{+}-\mathrm{N})}{HRT}$$

To test and verify the effect of the purified strain against thiourea shock load, reactor A and reactor B were used as control and experimental sets, respectively. BT1 was added to the experimental set as a bioaugment. Prior to the experiment, the reactors were inoculated with the same activated sludge, and the MLSS in the reactors were adjusted to the same level. The operation of the control reactor (reactor A) included seven continuous operational phases (Supplementary Table 1). In Phases II, IV, and VI, 2, 10, and 50 ppm thiourea were added to the synthetic wastewater, respectively. In Phases I, III, V, and VII, the influent was synthetic wastewater without thiourea. The operation of the experimental reactor (reactor B), similar to that of the control reactor, also included seven continuous operational phases (Supplementary Table 1). Actually, the impact of thiourea is also intermittent at municipal sewage, and the activated sludge also has the toxicity repair ability at some thiourea concentrations. So, the addition of synthetic wastewater was alternate to find out the suitable thiourea concentration which can lead the nitrification system to collapse. Strain BT1 in the logarithmic growth phase was continuously added to reactor B during Phases I to VII at a dose of 1% (volume BT1: volume influent). During the experiment, the MLSS in both reactors were controlled at 3,500–4,000 ppm, and the DO concentration was 2–4 ppm. The simulators did not include an anoxic section. They had an extended residence time in the sedimentation tank instead of in an anoxic section for in situ operability.

### High-Throughput Sequencing and Data Analysis

The microbial community in the activated sludge was investigated at phases I and VI in both reactors. DNA was extracted from each sludge sample using the E.Z.N.A. DNA Mag-Bind Soil Kit (Omega Biotek, Norcross, GA, USA) according to the manufacturer’s protocol and assessed by 1% agarose gel electrophoresis. The V3 and V4 regions of the bacterial 16S rRNA gene were amplified by PCR (94 °C for 3 min, 25 cycles at 94 °C for 30 s, 55 °C for 20 s, 72 °C for 30 s, and 72 °C for 5 min) using the primers 341F (5′-CCCTACACGACGCTCTTCCGATCTGCCTACGGGNGGCWGCAG-3′) and 805R (5-′GACTGGAGTTCCTTGGCACCCGAGAATTCCAGACTACHVGGGTATCTAATCC-3′). The sequencing library was constructed based on the PCR products and sequenced on the Illumina MiSeq platform at Sangon Biotech Technology Co., Ltd. (Shanghai, China). Raw sequence data were deposited in the NCBI Sequence Read Archive under the accession number PRJNA551309.

Operational taxonomic units (OTUs) were clustered with a distance limit of 0.03 (97% similarity) using the uclust-QIIME method [8, 18]. The number of OTUs was counted, and OTU abundance was calculated in R. The most abundant OTU sequence of each OTU was selected as the representative sequence and assigned to the taxonomic classification using the Ribosomal Database Project (RDP) Classifier with a confidence cutoff of 80%, applying SILVA software [29]. The average community compositions at the genus level were counted for each sample, and we performed principal component analysis including all OTUs. Additionally, alpha diversity (α-diversity) was calculated, including the rarefaction curve, sequencing coverage, Shannon diversity index, and Chao1 index, and the community composition of each sample was determined at the genus level.

### Restoration of Nitrification Ability of a Printed Circuit Board (PCB) Wastewater Treatment Plant

In late August/early September 2018, the nitrification system of a PCB wastewater treatment plant collapsed. When investigating the A/O system, it was found that the thiourea concentration of the influent was significantly high, suggesting that a high thiourea concentration was the main reason for the collapse. On the basis of the wastewater treatment process route in PCB production, we designed three process routes to quickly rebuild the nitrification system and evaluated them. The first was the activated carbon adsorption process, where we mixed 500 mL of effluent and 500 mL of activated sludge and added 0.01% activated carbon and 1% microbial bioaugmentation. The second was a combination of the Fenton method and a biological synergistic agent, where we first adjusted the effluent pH to 3.5 and added 0.5% H_2_O_2_ and 0.1% FeSO_4_·7H_2_O. After dissolving the FeSO_4_·7H_2_O, H_2_O_2_ was added, followed by mixing for 4 h and adjusting the pH to 9.0. The temperature of the reaction system was 25 °C. Finally, 500 mL of the supernatant was mixed with 500 mL activated sludge, and 1% of the biological synergist was added. The third consisted of activated sludge reflux combined with bioaugmentation; we added a 1% microbial bioaugmentation mixture with 150 mL of sludge and 50 mL of physicochemical effluent. After 2 h, 600 mL of the mud was mixed with physicochemical effluent at a ratio of 1:10 for 1 h, and 60 mL of the supernatant was added back (Tables 2, 3, and 4). The bioaugmentation mix for these three routes was provided by this study and was mainly composed of *Pseudomonas* BT1, with a strain concentration of 1.57 ± 0.5 × 10^9^ cells/mL. We inoculated the bioaugmentation mix in a culture medium containing 20 ppm thiourea and 100 ppm ammonia nitrogen at a rate of 1% at 30 °C and 160 r/min for 48 h, representing the experimental group. The culture medium without thiourea was set as the control group to confirm the tolerance of the bioaugmentation mix to thiourea.

**Table 2:** Degradation of ammonia in the physicochemical effluent by activated carbon adsorption

Time/h	1% microbial agent		1% activated carbon		1%activated carbon+1% microbial agent	
	NH4+-N/(mg·L^−1^)	pH	NH4+-N/(mg·L^−1^)	pH	NH4+-N/(mg·L^−1^)	pH
1	26.38	7.75	24.34	7.75	25.61	7.71
5	22.85	7.82	20.5	7.82	22.71	7.76
17	19.54	7.75	22.75	7.75	11.85	7.61

**Table 3:** Degradation of ammonia in the physicochemical effluent by the combination of Fenton and a biological synergistic agent

Time/h	1% microbial agent		Fenton treatment		Fenton treatment+1%microbial agent	
	NH4+-N/(mg·L^−1^)	pH	NH4+-N/(mg·L^−1^)	pH	NH4+-N/(mg·L^−1^)	pH
1	26.38	7.75	38.03	7.55	31.54	7.52
5	22.85	7.82	41.2	7.51	42.89	7.55
17	19.54	7.75	33.75	7.32	19.53	7.25

**Table 4:** Degradation of ammonia nitrogen in effluent by activated sludge reflux adsorption

Time/h	1% microbial agent		activated sludge reflux		activated sludge reflux+1% microbial agent	
	NH4+-N/(mg·L^−1^)	pH	NH4+-N/(mg·L^−1^)	pH	NH4+-N/(mg·L^−1^)	pH
1	26.38	7.75	22.71	7.78	37.63	7.6
5	22.85	7.82	23.71	7.53	30.12	7.6
17	19.54	7.75	20.89	7.63	0.41	7.2

## Results

### t Kitchen Sewage Sludge Restored the Nitrification System of a 6000 t/d Municipal Wastewater Treatment Plant

**Fig. 1:**
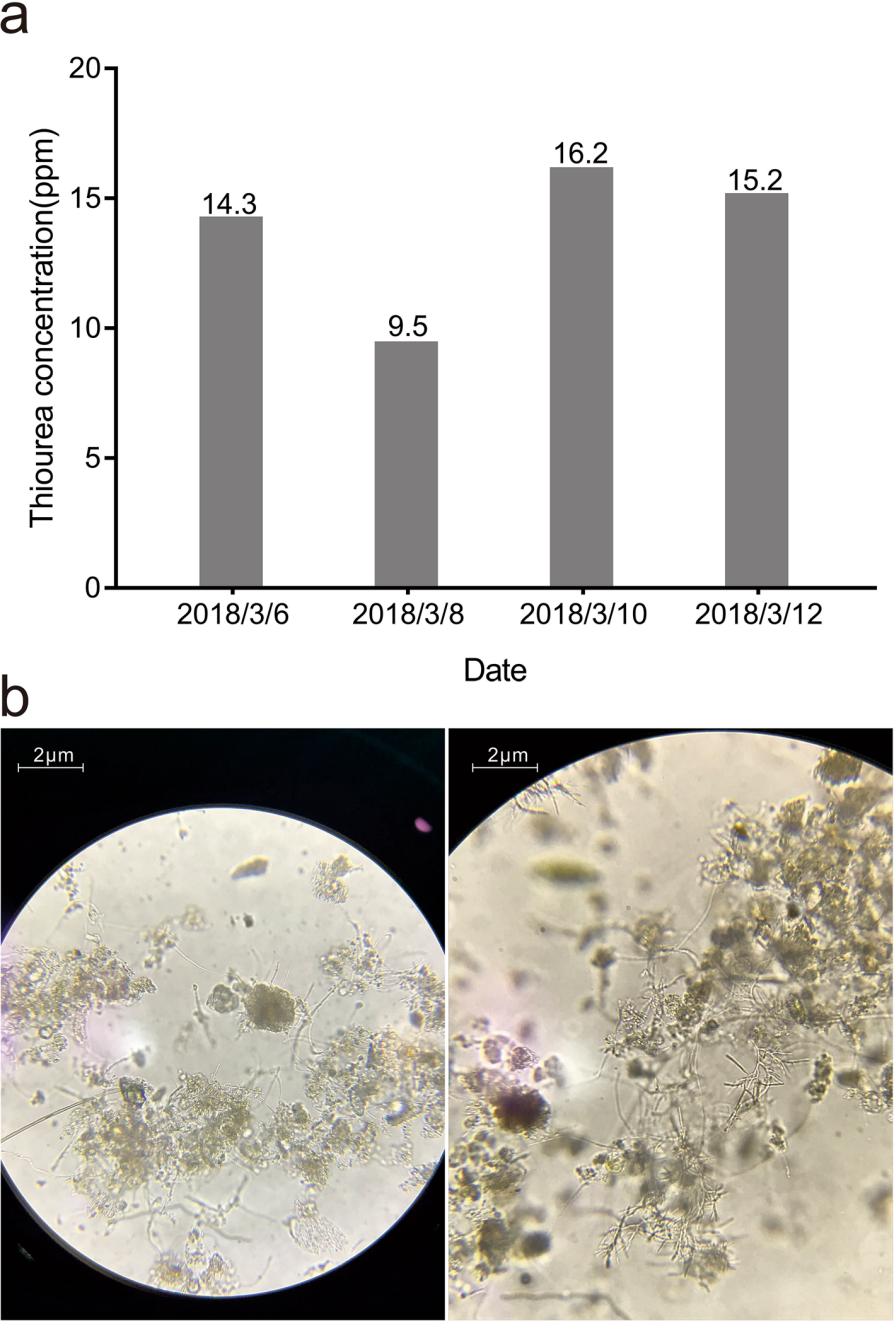
**a** Thiourea concentrations of the influent (INF) and effluent (EFF) water of the 60,000-t/d sewage treatment plant (ND, not detected). **b** Microscopic examination of the activated sludge after toxic shock, showing the reproduction of filamentous bacteria

Microscopic examination showed filamentous bacterial reproduction (Fig. 1b). After excluding the effects of heavy metals, temperature, reflux, and other factors, thiourea toxicity could be preliminarily determined. The thiourea content in the influent and effluent water was 5–15 ppm (Fig. 1a), which was considerably above the tolerated level (1 ppm) [36, 37]. Data monitoring and microscopic examination of microorganisms confirmed that the significant reduction in the nitrification removal rate was caused by sulfur compounds in the influent water. Although these compounds have a small impact on COD, they are toxic to nitrifying and denitrifying microorganisms.

**Fig. 2:**
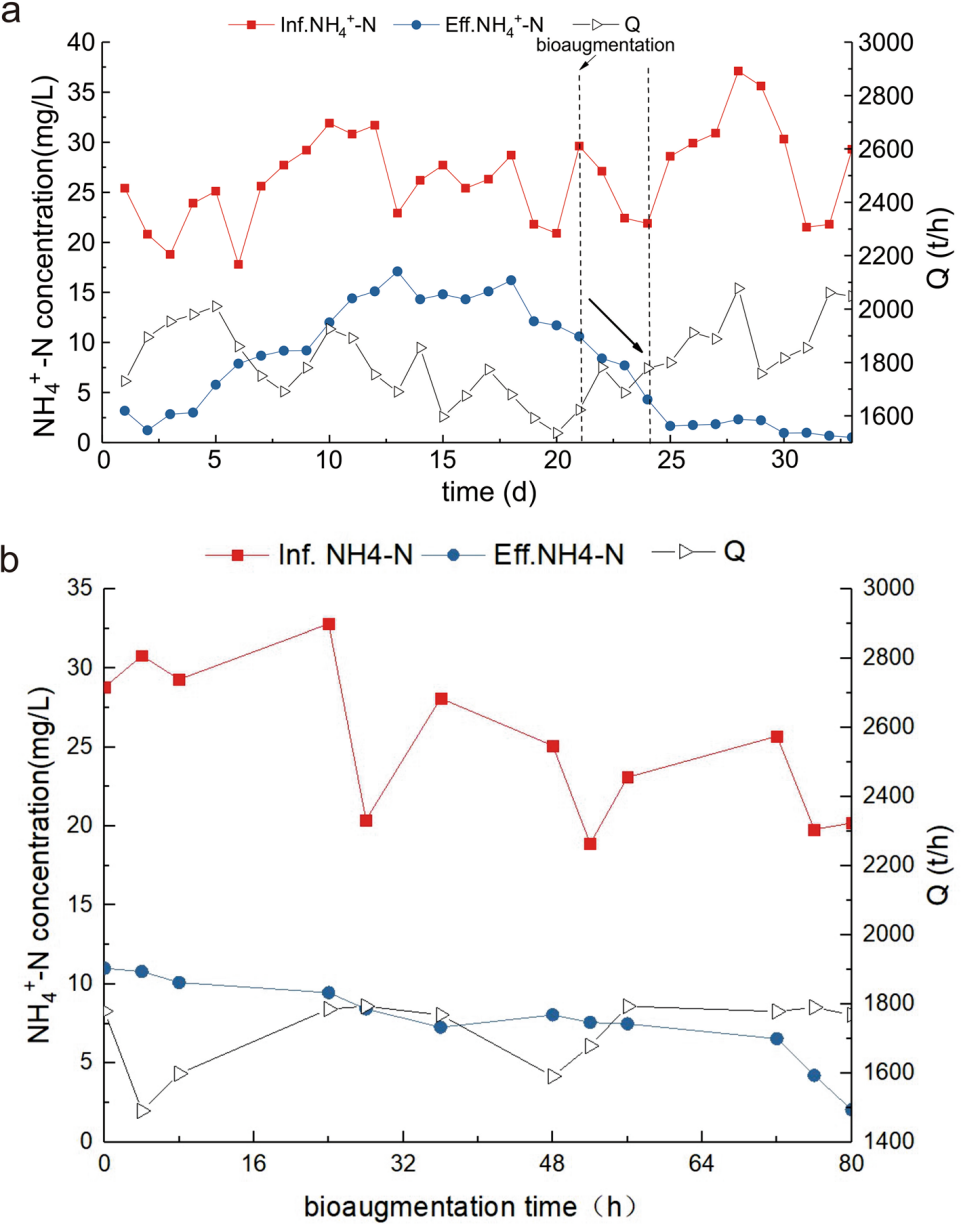
Recovery of the investigated nitrification system over time. **a** Changes in NH_4_^+^-N concentrations of the influent (INF) and effluent (EFF) water over time. **b** Changes in NH_4_^+^-N concentrations of the INF and EFF water during bioaugmentation time

The removal efficiency of the investigated kitchen sewage treatment system for ammonia nitrogen and total nitrogen was 5–10 times that of the 60,000 t/d municipal wastewater treatment plant (Table 1). The ammonia nitrogen level of the effluent decreased rapidly from 15 ppm to less than 5 ppm (emission standard is less than 5 ppm) after 48 h and to a level below 2 ppm after 96 h, indicating that the nitrification system was successfully restored (Fig. 2).

### Strain Isolated from 10 t Kitchen Waste Sludge Identified as *Pseudomonas BT1*

The sludge in the high-efficiency denitrification system was purified to isolate bacterial stains for 16S rDNA identification. After the first purification, the obtained HNB represented a colony composed of six to seven species, dominated by *Pseudomonas* (accounting for approximately 80%), followed by *Rhizobium* and *Stenotrophomonas*, while the other strains only accounted for approximately 5% of the total bacteria (Supplementary Table 2). The bacterial colony was further diluted and streaked on plates in more than 10 rounds to isolate the pure strain, and its 16S rDNA amplification sequencing data were uploaded to the NCBI database under the accession number PRJNA551309. This strain has not been reported before based on the NCBI database and was named *Pseudomonas* BT1.

### Pure Strain *Pseudomonas* BT1 Could Grow and Remove Ammonia Nitrogen Under 500 ppm Thiourea Shock

**Fig. 3:**
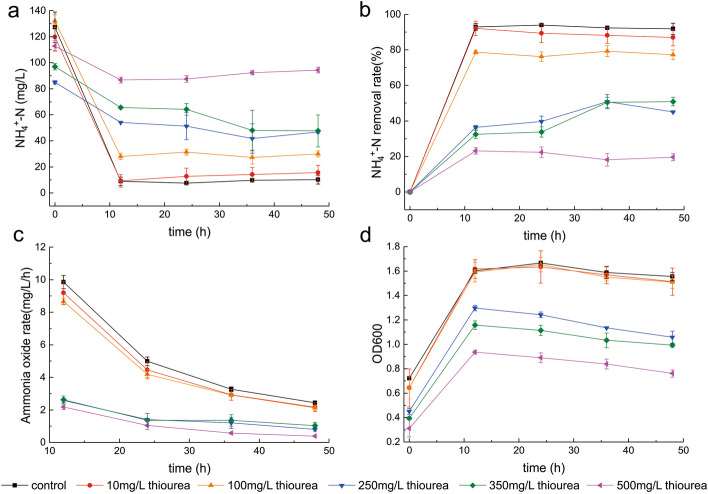
*Pseudomonas* BT1 under six thiourea concentrations (0, 10, 100, 250, 350, and 500 ppm). **a** NH4+-N concentration; **b** NH4+-N removal rate; **c** ammonia oxide rate; and **d** OD600 changes. Data points and error bars represent the average and standard deviations of the three replicates, respectively

Thiourea, as an inhibitor of proprietary nitrification, significantly impedes the conversion of ammonia to nitrite [37], [34]. We performed a shake flask experiment using six groups with different thiourea concentrations (0, 10, 100, 250, 350, and 500 ppm) to evaluate the degradation efficiency of *Pseudomonas* BT1 under thiourea shock,the 0 ppm thiourea group served as the control. As shown in Fig. 3a, the NH_4_^+^-N removal rate decreased with increasing thiourea concentration, and the changes in AOR were similar to those in NH_4_^+^-N removal (Fig. 3c). When the thiourea concentration was 10 ppm, the NH_4_^+^-N removal rate was similar to that of the control but decreased suddenly when the thiourea concentration was 100 ppm. The NH_4_^+^-N removal rate decreased from 92 to 78% at thiourea levels above 250 ppm, indicating that the ammonia oxidation efficiency of BT1 was affected by high thiourea concentrations, although the impact was limited (Fig. 3b). At thiourea concentrations of 0, 10, or 100 ppm, the trend of OD600 was similar to that of the BT1 growth rate, showing that BT1 can grow at high thiourea levels (Fig. 3d). In general, thiourea concentrations below 100 ppm could not inhibit the growth and activity of *Pseudomonas* BT1, suggesting that it can be used to improve the nitrification efficiency of sewage treatment plants after thiourea shock.

### *Pseudomonas* BT1 Mixed with Activated Sludge Could Remove Ammonia Nitrogen Under 10 ppm Thiourea Shock

**Fig. 4:**
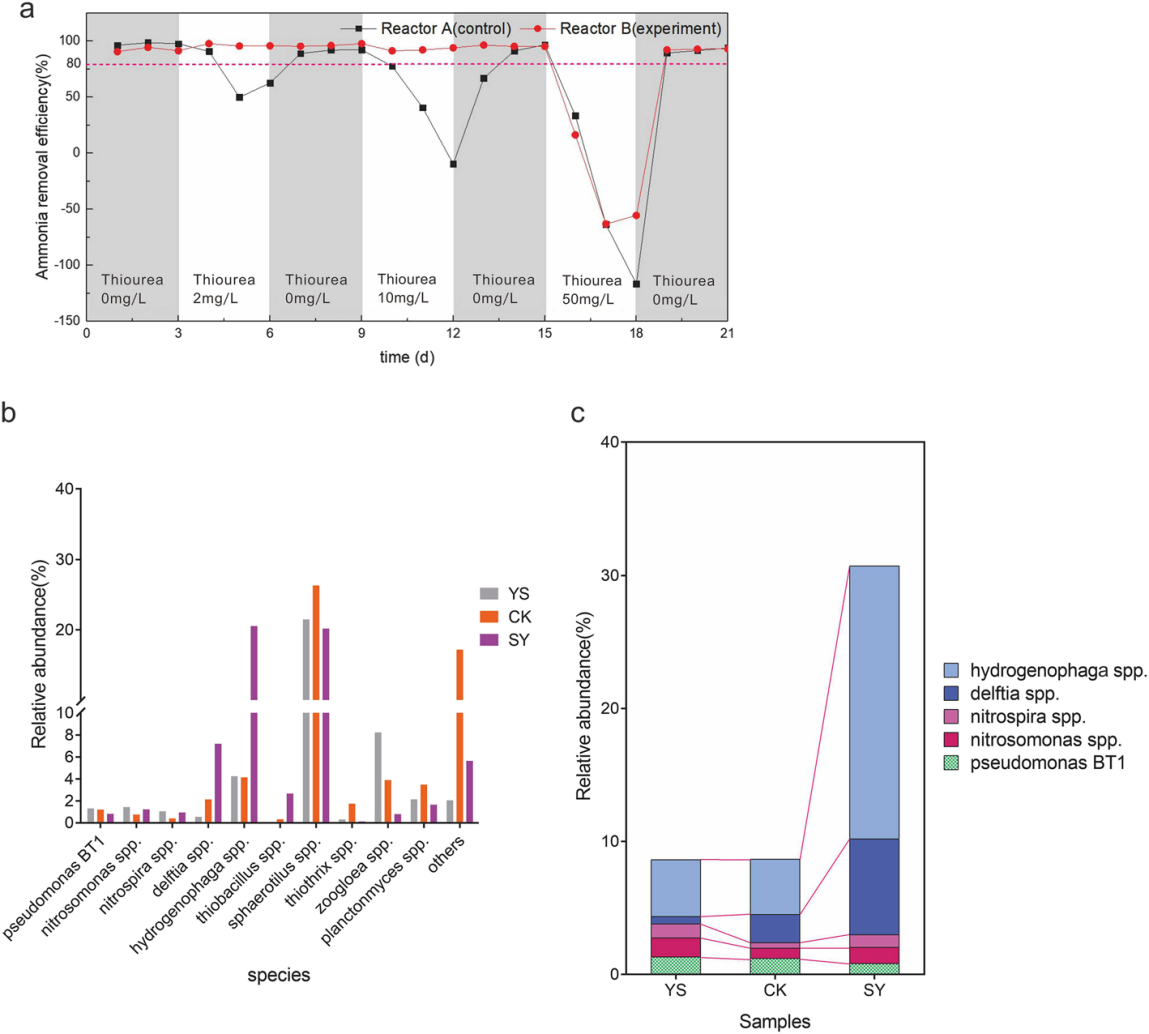
**a** Ammonia removal efficiencies of the reactors at different thiourea concentrations. **b** Microbial community composition of the activated sludge at the genus level in the initial and final stages. **c** The dominant genera with significant differences in the initial and final stages of the two reactors (★YS: initial activated sludge from WWTPs; CK: activated sludge at Phase VII of reactor A; and SY: activated sludge at Phase VII of reactor B)

To verify the bioaugmentation of BT1 under thiourea shock, one reactor served as the experimental group (reactor B) and performed the bioaugmentation, and the other was the control group (reactor A). In phase I (1–3 days), the thiourea concentration was 0 ppm, and the ammonia nitrogen removal rates were 97 and 91% in the control and experimental groups, respectively. When the thiourea concentration increased to 2 ppm (phase II, 4–6 days), the ammonia removal rate in the control group rapidly decreased to 49% and then slowly recovered to 91% until the thiourea in the influent water reached 0 ppm. In contrast, the ammonia removal rate of the experimental group increased to 96% when the thiourea concentration was 2 ppm below the bioaugmentation level of *Pseudomonas* BT1. When the concentration of thiourea increased to 10 ppm, the ammonia removal rate of the experimental group remained at 92%, whereas that of the control group decreased to 0%. Based on the thiourea resistance of *Pseudomonas* in pure medium, the ammonia nitrogen removal rate decreased to 78% when the thiourea concentration was 100 ppm. In contrast, the ammonia nitrogen removal rates of the experimental group and control group both decreased to 0% when the concentration of thiourea increased to 50 ppm, while the ammonia removal rate of the experimental group recovered more rapidly than that of the control group (Fig. 4a). When the ammonia nitrogen concentration of the wastewater was above 5 ppm or the removal rate was below 80%, the efficiency of the nitrification system was low, and the ammonia nitrogen level exceeded the threshold. Because of the bioaugmentation of *Pseudomonas* BT1 in reactor B, the ammonia nitrogen removal rate could remain above 90% at a thiourea concentration of up to 10 ppm. At influent thiourea concentrations of more than 2 ppm over 48 h, ammonia nitrogen exceeded the threshold,at influent thiourea concentrations of more than 10 ppm over 48 h, the nitrification system collapsed. The monitoring data of the simulator were consistent with the operation of the sewage treatment plant.

### Relative Abundance of HNB and Sulfur-Oxidizing Bacteria Increased in Activated Sludge Containing *Pseudomonas* BT1

The microbial compositions of activated sludge in the reactors were investigated using high-throughput sequencing. After quality filtering, a total of 505,362 good-quality sequences with an average length of 375 bp from all reactor samples were obtained. All high-quality sequences were assigned to 7,563 OTUs with 97% similarity (Supplementary Figure 2). The microbial richness (Chao) and diversity (Shannon) indices of microbial communities in the two reactors decreased significantly during thiourea shock loading. Figure 4b shows the microbial community compositions of the activated sludge in the experimental group and the control group; only the 10 core genera with a relative abundance above 1% are shown. In the initial and final stages, *Sphaerotilus*, *Hydrogenophaga*, and *Delftia* were the dominant genera in both reactors. In the experimental group, the relative abundance of *Hydrogenophaga* increased from 4.25 to 20.5%, whereas in the control group, it decreased from 4.25 to 4.14%. *Hydrogenophaga* is an autotrophic denitrifying microorganism closely related to nitrogen removing bacteria. In our study, the relative abundances of *Nitrosomonas* and *Nitrospira* in the experimental group were 0.93 and 0.13%, respectively, which were higher than those of the control group (0.4 and 0.03%, respectively) (Fig. 4c). HNB in reactors, such as *Delftia* and *Pseudomonas*, also played an important role in nitrogen removal. For example, the relative abundance of *Delftia* in the experimental group and the control group increased from 0.5% in phase I to 7.1 and 2.1%, respectively. In phase VII, the relative abundance of *Pseudomonas* in the experimental group decreased from 1.2 to 0.4%, whereas in the control group, it only decreased to 1.0%.

### *Pseudomonas* BT1 Restored the Nitrification System of a Printed Circuit Board (PCB) Wastewater Treatment Plant

The activated carbon adsorption results are shown in Table 2. When only 1% *Pseudomonas* BT1 was added to the physicochemical effluent, the ammonia degradation rate was 25.9% after 17 h. Ammonia degradation was 6.5% when the physicochemical effluent was treated only with activated carbon, whereas it increased to 53.7% after using activated carbon adsorption for 17 h. The results of the combination of the Fenton method and a biological synergistic agent are shown in Table 3. The physicochemical effluent was treated by the Fenton method and combined with a biological synergistic agent; after 17 h, the ammonia nitrogen degradation rate reached 38.07%, with an increase of 12.17% compared with the control group. Compared with the activated carbon adsorption process, the effect of this method is not satisfactory. When we performed the assessment of activated sludge reflux combined with bioaugmentation, we placed *Pseudomonas* BT1 into the aerobic tank to encourage propagation. After adaptation to the sewage treatment system, the remaining sludge was returned to the secondary physicochemical treatment tank, and biological detoxification was carried out by the activated sludge. After detoxification, the sewage entered the biochemical system, and the biological synergist could further exert its synergistic effect (Table 4). The combination of activated sludge reflux and microbial synergist could remove ammonia from physicochemical water within 17 h, and the ammonia degradation rate reached 98.9%.

**Fig. 5:**
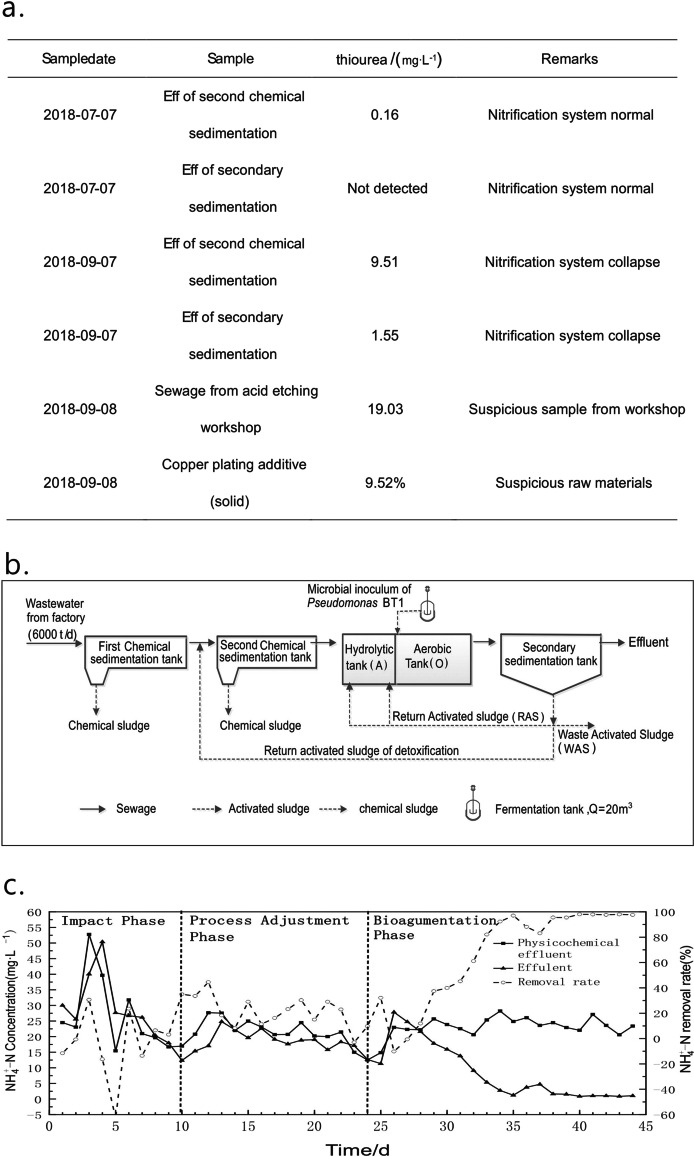
Bioaugmentation with the use of *Pseudomonas* BT1 in PCB wastewater. **a** Thiourea detection of biochemical system effluent before and after the collapse; **b** A/O activated sludge reflux combined with bioaugmentation process; **c** change of ammonia nitrogen degradation rate in. each unit of A/O system

Activated sludge reflux combined with bioaugmentation was used for field operation in a 6,000-t/d PCB wastewater treatment system, and the ammonia content of the aerobic tank decreased after the modification of the reflux pipe and the addition of the biological synergist (Fig. 5a). The ammonia degradation rate of the A/O biochemical system was −20–20% during the PCB wastewater shock load (Fig. 5b). With improved detoxification, the ammonia nitrogen degradation rate significantly increased to 40–60% within 7 days. After approximately 15 days, the nitrification system returned to normal, and the degradation rate stabilized at more than 95% (Fig. 5c).

## Discussion

Table 5 shows the major genera and their respective representative species involved in bioaugmentation, along with their relative abundances from the beginning to the end of the experiment in both reactors. The ammonia removal efficiency of the control group was completely inhibited by thiourea, caused by the inhibition of *Nitrosomonas* (representative strain of AOB). Since ammonia degradation is mostly accomplished by AOB and NOB [7], the increase in filamentous bacterial abundance could lead to activated sludge bulking, affecting sludge activity. Filamentous bacteria played a dominant role in the activated sludge of the control reactor. Based on our results, it is necessary to re-evaluate the function of *Pseudomonas* in the activated sludge of sewage treatment systems, especially the redistribution of ammonia-oxidizing bacteria under toxic shock. In a previous study, K-strategists and R-strategists were common in the activated sludge community [26]. A study based on a correlation analysis of an activated sludge network revealed nonrandom taxonomic correlations among global functional bacteria [15]. The core bacteria were divided into K-strategists and R-strategists, and the R-strategists could reduce the impacts of toxic substances and were more efficient in the continuous activated sludge system. However, with the addition of *Pseudomonas* BT1 for bioaugmentation, the large number of *Pseudomonas* BT1 cells did not play a dominant role. In previous studies, the introduced strain for bioaugmentation dominated the activated sludge [4], [24]. Obviously, the “K and R-strategies” hypothesis cannot fully explain the community changes in the reactor.

**Table 5:** Abundances of the major genera in the two reactors and variations in abundance patterns

Number	Typical classification	Genera	Abundance (%)			Abundance shift	
			YS	CK	SY	CK-VS-YS	SY-VS-YS
1	BT1	*Pseudomonas BT1*	1	——	——	——	——
2	AOB	*Nitrosomonas* spp.	1.4	0.8	1.2	↓a	↓
3	NOB	*Nitrospira* spp.	1.1	0.4	0.9	↓	↓
4	HNB	*Delftia* spp.	0.6	2.1	7.2	↑	↑↑b
5		*Pseudomonas* spp.	1.3	1.2	0.8	↓	↓
6	Filamentous	*Thiothrix* spp.	0.3	1.7	0.1	↑	↓
7		*Sphaerotilus* spp.	21.5	26.3	20.2	↑	↓
8	others	*Planctomyces* spp.	2.1	3.5	1.6	↑	↓
9		*Hydrogenophaga* spp*.*	4.3	4.1	20.5	↓	↑↑↑c
10		*Zoogloea* spp.	8.2	3.9	0.8	↓↓	↓↓
11		*Thiobacillus* spp.	0	0.3	2.7	↑	↑↑

a↑/↓: <2%; b↑↑/↓↓:2-5%; c: ↑↑↑/↓↓↓: >5%

In both reactors, the relative abundances of *Thiobacillus* and *Thiothrix* increased; both species used S^2−^ as the substrate. Thiourea can be degraded into S^2−^ by other factors; the resulting NH4^+^-N can be used by AOB and HNB [28]. *Pseudomona*s BT1 might produce a secondary metabolite that could stimulate the growth of *Hydrogenophaga*, accelerate the degradation of thiourea, and relieve the inhibition of AOB by thiourea under the action of other microorganisms. These excessive secondary metabolites might inhibit or even destroy *Pseudomonas* BT1, causing a decrease in BT1 abundance. The functional community in the activated sludge was divided into four categories, namely, AOB, NOB, HNB, and other microorganisms (Supplementary Figure 3). When the activated sludge was shocked by thiourea, the control group lost its nitrification stability due to the impacts on AOB, while the experimental group could maintain its ammonia removal efficiency because of the addition of *Pseudomonas* BT1. The abundance of *Pseudomonas* BT1 decreased in reactor B, whereas that of *Hydrogenophaga* and *Thiobacillus* increased. The “intermediate product” hypothesis could explain the contradiction between the nongrowth of the strain and the bioaugmentation effect in the presence of *Pseudomonas* BT1.

The application of the bioaugmentation mix was limited by several factors, including the adaptability of the inoculated strains [26], competition between inoculated strains and native organisms, insufficient substrate nutrition, and capture of protozoa [5, 6]. The most important factors were the quantity and quality of the core strains applied. Increasing the inoculated strain number could prevent the loss of microorganisms from the reactor, but excessive inoculated strains would have a negative impact on the structure and function of the local microbial community [27]. In this study, when *Pseudomonas* BT1 was added to the reactor, the relative abundance of *Pseudomonas* in the reactor decreased, and *Pseudomonas* BT1 did not occupy a dominant position in the community. *Pseudomonas* BT1 had no negative impact on the activated sludge system and enhanced the stability of the nitrification system. To observe the continuous changing of the microbial community, more phases can be selected to do the comparison in future.

The 16S rRNA analysis can illustrate the composition and relatively abundance of microbiome in the sample, but cannot supply more details about their functions. On the other side, because of the functional diversity of HNB, it is also difficult to achieve the functions of *Pseudomonas* BT1 in the meta samples. The genome and transcriptome analysis on the isolated *Pseudomonas* BT1 is necessary in the future to investigate genes related to toxic substance metabolic pathways and bioenhancement performance, giving attention to the symbiosis or competition between traditional ANB and *Pseudomonas* in activated sludge systems.

## Conclusions

Based on successful bioaugmentation, a wild heterotrophic nitrifying strain, *Pseudomonas* BT1, was obtained and isolated from the high-efficiency nitrification sludge of a kitchen sewage treatment plant. Physiological and biochemical analyses of *Pseudomonas* BT1 showed that the pure strain could rapidly proliferate and maintain ammonia oxidation activity at thiourea concentrations of 10–500 ppm. Thiourea shock (2–10 ppm) was simulated, and the ammonia removal efficiency decreased to 0% in the control group, while it remained at 90% in the experimental group after adding 1% *Pseudomonas* BT1. When the relative abundance of BT1 was high, the abundance of the strains involved in ammonia oxidation and sulfur oxidation, including *Hydrogenophaga*, *Delftia*, and *Thiobacillus*, increased. *Pseudomonas* BT1 was used in a 6,000-t/d PCB wastewater treatment system, the nitrification system returned to normal in 15 days, and the degradation rate stabilized at more than 95%.

## Supplementary Information

Below is the link to the electronic supplementary material.

Supplementary file1 (PNG 106 KB)

Supplementary file2 (JPG 4865 KB)

Supplementary file3 (JPG 1861 KB)

Supplementary file4 (XLSX 11 KB)

Supplementary file5 (XLSX 11 KB)
