# Supplementary figures and images for: Wild Heterotrophic Nitrifying Strain Pseudomonas BT1 Isolated from Kitchen Waste Sludge Restores Ammonia Nitrogen Removal in a Sewage Treatment Plant Shocked by Thiourea
Source: Appl Biochem Biotechnol. 2022 Mar 16;194(7):2901–18. doi: 10.1007/s12010-022-03850-7 (PMC9205789; doi:10.1007/s12010-022-03850-7)

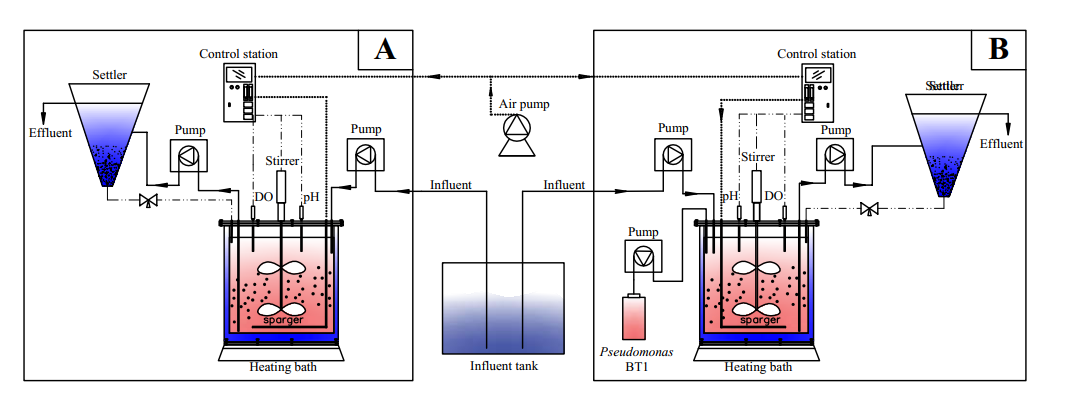

Supplement: Supplementary file 1 — Supplementary file1 (PNG 106 KB) [file 12010_2022_3850_MOESM1_ESM.png]

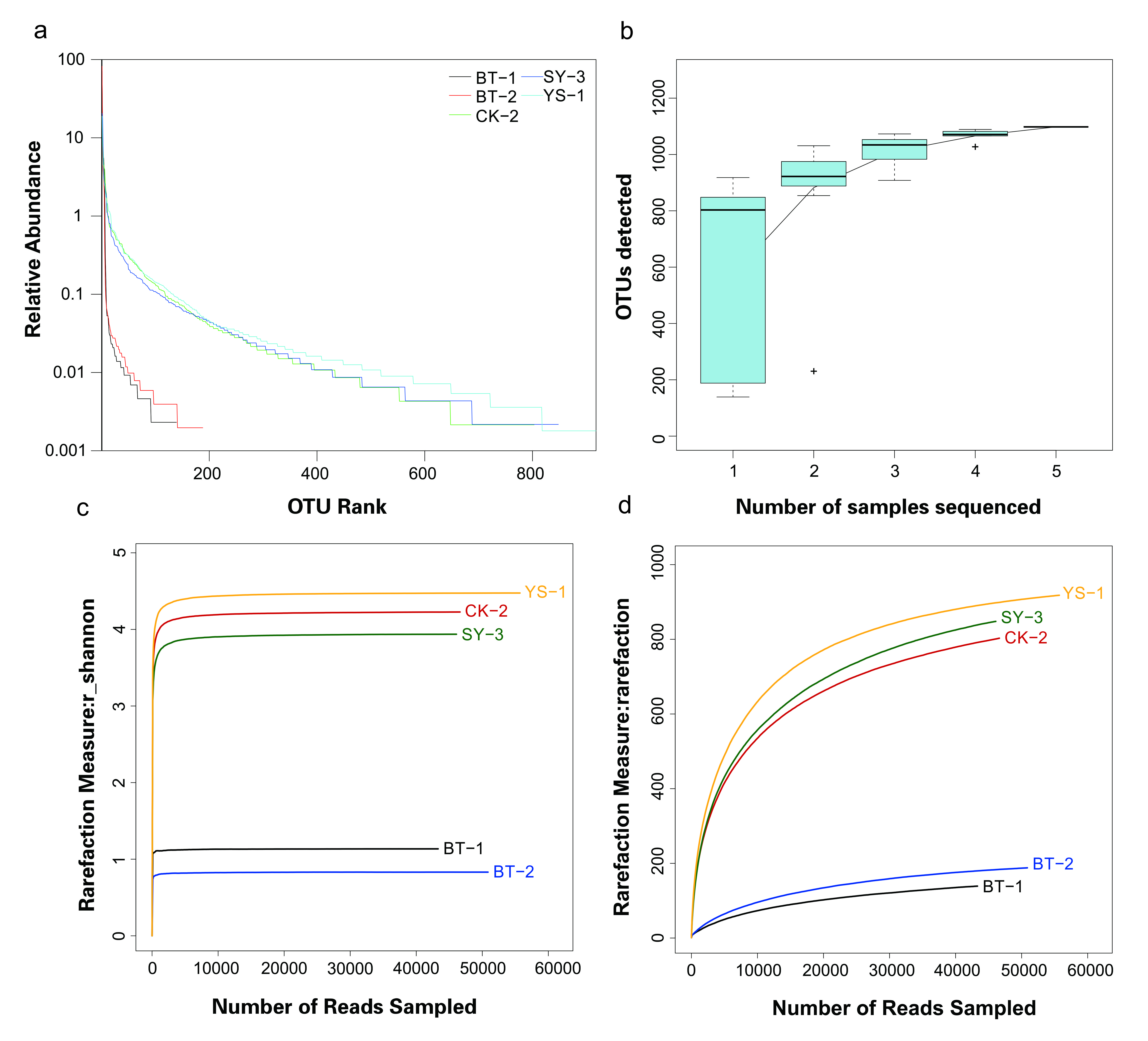

Supplement: Supplementary file 2 — Supplementary file2 (JPG 4865 KB) [file 12010_2022_3850_MOESM2_ESM.jpg]

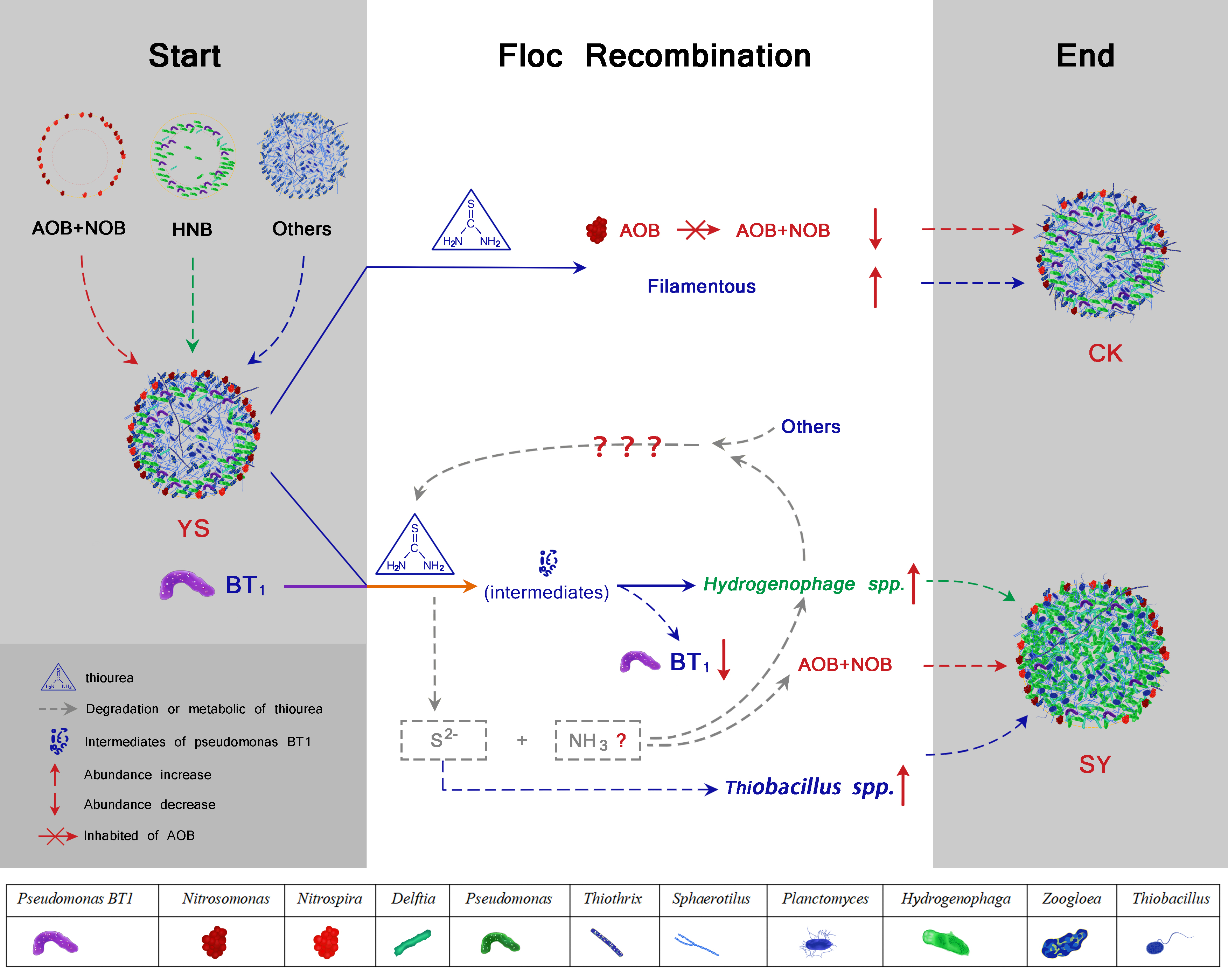

Supplement: Supplementary file 3 — Supplementary file3 (JPG 1861 KB) [file 12010_2022_3850_MOESM3_ESM.jpg]
